# Progesterone receptor (PR) intra‐tumor heterogeneity in premenopausal breast cancer: A secondary analysis of a randomized trial

**DOI:** 10.1002/ijc.70209

**Published:** 2025-10-24

**Authors:** Oscar Danielsson, Huma Dar, Gizeh Perez‐Tenorio, Anna Nordenskjöld, Christina Yau, Christopher C. Benz, Laura J. Esserman, Bo Nordenskjöld, Olle Stål, Tommy Fornander, Johan Hartman, Nicholas P. Tobin, Annelie Johansson, Linda S. Lindström

**Affiliations:** ^1^ Department of Oncology and Pathology Karolinska Institutet Stockholm Sweden; ^2^ Breast Center, Karolinska Comprehensive Cancer Center, Karolinska University Hospital Stockholm Sweden; ^3^ Department of Biomedical and Clinical Sciences and Department of Oncology Linköping University Linköping Sweden; ^4^ Institution of Clinical Sciences, Department of Oncology Sahlgrenska Academy at Gothenburg University Gothenburg Sweden; ^5^ Buck Institute for Research on Aging Novato California USA; ^6^ Department of Surgery University of California San Francisco San Francisco California USA; ^7^ Department of Medicine University of California San Francisco San Francisco California USA

**Keywords:** breast cancer, immunohistochemistry, long‐term risk, premenopausal, progesterone receptor heterogeneity

## Abstract

Premenopausal breast cancer patients have an increased risk of distant recurrence, but their long‐term risk remains unclear. Notably, over 90% of estrogen receptor (ER) positive tumors in premenopausal patients are also progesterone receptor (PR) positive, compared to 70% in postmenopausal patients. We aimed to determine whether PR intra‐tumor heterogeneity influences long‐term risk of distant recurrence and endocrine therapy benefit in premenopausal breast cancer patients. We conducted a secondary analysis of the Stockholm tamoxifen (STO‐5) randomized controlled trial (1990–1997) with 20‐year complete follow‐up, including 924 premenopausal women with operable breast cancer in Stockholm, Sweden. Patients were randomized to 2 years of adjuvant endocrine therapy or control, with lymph node‐positive patients receiving standard chemotherapy (CMF). Tumor blocks were available for 731 patients. PR intra‐tumor heterogeneity was assessed by measuring variation in PR immunohistochemical staining intensity in whole tumor slides and was categorized as high or low for 520 ER‐positive/PR‐positive patients. Distant recurrence‐free interval (DRFI) by PR heterogeneity was analyzed using Kaplan–Meier, multivariable Cox proportional‐hazards regression, and multivariable time‐varying flexible parametric modeling. We found PR intra‐tumor heterogeneity significantly impacted 20‐year DRFI (log‐rank *p* = .002). Patients with high intra‐tumor heterogeneity had a significantly increased risk of distant recurrence, compared to patients with low heterogeneity (hazard ratio [HR] = 1.42; 95% CI, 1.02–1.96). Similar results were observed in HER2‐negative patients. Patients with high PR heterogeneity significantly benefited from endocrine therapy (HR = 0.41; 95% CI, 0.24–0.71). These findings suggest that premenopausal patients with high PR heterogeneity have increased long‐term risk but significantly benefit from endocrine therapy.

## Graphical abstract

What's new?

Progesterone receptor (PR) expression in breast cancer is influenced by menopausal status and PR positivity is more common in premenopausal women. The influence of PR intra‐tumor heterogeneity on distant recurrence risk in premenopausal women, however, remains unknown. In this investigation, variations in PR expression within breast tumors, measured by immunohistochemical staining intensity in tumor tissue, were associated with increased long‐term risk of distant recurrence. Patients with high PR intra‐tumor heterogeneity, however, also benefited from endocrine treatment. The findings highlight the feasibility of assessing PR intra‐tumor heterogeneity by immunohistochemistry and offer insight into long‐term recurrence risk in premenopausal breast cancer patients.

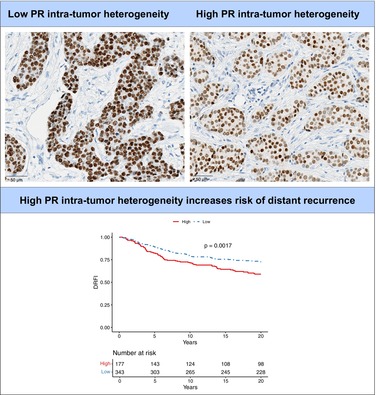

**Abbreviations**
BCSS: breast cancer specific survival
CI: confidence interval
CMF: cyclophosphamide, methotrexate and fluorouracil
DRFI: distant recurrence‐free interval
ER: estrogen receptor
HER2: Human Epidermal Growth Factor Receptor 2
HR: hazard ratio
IHC: immunohistochemistry
PR: progesterone receptor

## 1 INTRODUCTION

The heterogeneity of breast cancer is evident both in its aggressiveness and potential time to distant metastases, which ranges from months to decades following primary breast cancer diagnosis. Importantly, there is an increased risk of fatal breast cancer for premenopausal as compared to postmenopausal women,^1^, ^2^, ^3^, ^4^ with evidence suggesting that the diseases are biologically distinct entities.^5^, ^6^, ^7^, ^8^ Given these age‐related differences and the fact that premenopausal patients are diagnosed early in life, it is crucial to identify tumor characteristics contributing to increased long‐term risk in this patient group.

It is well‐known that the estrogen receptor (ER) plays a vital role in modulating growth and differentiation in breast cancer. Although less understood, progesterone receptor (PR) expression is believed to influence ER functionality.^9^ The proportion of ER‐positivity is similar across premenopausal and postmenopausal patients, with approximately 80% of tumors expressing ER, whereas interestingly PR‐positivity varies by menopausal status in women with ER‐positive tumors. Among premenopausal women diagnosed with ER‐positive breast cancer, around 90% are also PR‐positive, compared to approximately 70% in postmenopausal women, as seen in our and other studies.^10^

Patients with ER‐positive tumors have a substantial long‐term risk of distant recurrence,^11^, ^12^, ^13^ with at least half of all distant recurrences occurring beyond 5 years after primary diagnosis.^14^ The underlying biology behind this long‐term risk in ER‐positive breast cancer is yet to be understood. One proposed mechanism is tumor cell dormancy, referring to a state where tumor cells are quiescent but can become active once certain microenvironmental conditions are favorable.^14^ Present‐day clinical decisions for ER‐positive patients rely on the clinically used tumor characteristics including the breast cancer markers: PR, human epidermal growth factor receptor 2 (HER2), and proliferation marker Ki‐67. However, these markers only partially capture the full complexity of the disease, one reason for this being variability within the tumor. This intra‐tumor heterogeneity can influence the risk of distant recurrence, as corroborated by our findings and those of other researchers.^15^, ^16^

In this study we aimed to determine whether intra‐tumor heterogeneity of PR influences the long‐term risk of distant recurrence and endocrine therapy benefit in premenopausal women with ER‐positive disease, using the Stockholm tamoxifen trial (STO‐5) which has now reached 20 years of complete follow‐up. Our assessment of intra‐tumor heterogeneity uses standard immunohistochemistry (IHC) staining of PR, and expression intensity was annotated by breast cancer pathologists. A better understanding and identification of new tumor markers associated with long‐term risk of fatal breast cancer are essential for improving clinical management, especially for premenopausal women given their worse prognosis but otherwise long life expectancy.

## 2 MATERIALS AND METHODS

### 2.1 Patients

The Stockholm tamoxifen trial (STO‐5) enrolled 924 premenopausal patients diagnosed with invasive operable breast cancer, between May 1990 and January 1997.^17^, ^18^, ^19^, ^20^, ^21^, ^22^ After surgery, the primary tumors were formalin‐fixed paraffin‐embedded (FFPE) and archived. Premenopausal status was defined as patients having a menstrual cycle within 6 months of diagnosis. Patients were randomized to either receive adjuvant endocrine therapy (2 years of tamoxifen, goserelin, combined goserelin and tamoxifen) or no adjuvant endocrine therapy (control). The randomization was stratified by lymph node status and lymph‐node‐positive patients received further standard adjuvant chemotherapy (CMF: cyclophosphamide, methotrexate and fluorouracil; see Supplementary Methods). The randomization was done centrally, and allocated treatment was disclosed to patients and physicians after randomization. The primary endpoints were overall survival and recurrence‐free survival.

Each resident in Sweden is assigned a unique personal identity number, which makes it possible to link various personal data records. In this study, follow‐up until December 31, 2016, was possible using Swedish high‐quality national and regional registries of high validity and essentially complete coverage.^23^, ^24^, ^25^

### 2.2 Immunohistochemistry

**FIGURE 1:**
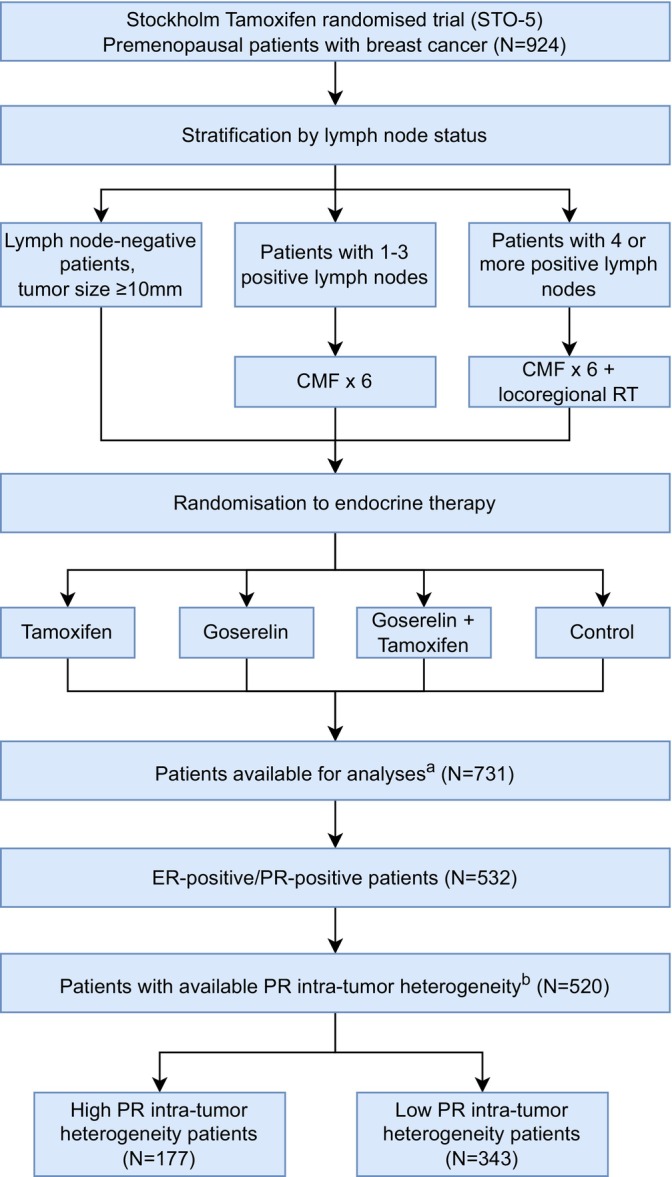
Consort diagram of the Stockholm tamoxifen (STO‐5) randomized clinical trial. ^a^Out of the original STO‐5 cohort of 924 premenopausal patients 731 patients had available paraffin embedded tumor blocks. ^b^520 out of 532 patients with ER‐positive/PR‐positive breast cancer had information on PR intra‐tumor heterogeneity.

Primary tumor FFPE blocks with sufficient invasive tumor cells were available for 731 patients (Figure 1). The distribution of patient and tumor characteristics was similar for this subset of 731 patients as compared with the 924 patients originally enrolled (Supplementary Table 1). IHC analysis was performed in 2020 in collaboration with the Karolinska University Hospital Pathology Department, using standardized clinical protocols. The percentage of cancer cells positive for ER, PR, HER2 and Ki‐67 was scored by experienced breast cancer pathologists. In addition, the PR staining intensity (0, 1+, 2+, 3+) together with the proportion of tumor cells for each staining intensity was also assessed by the pathologists. According to the Swedish National Guidelines, ER‐positivity and PR‐positivity were defined by a threshold of 10% or greater, HER2‐positivity was defined as intensity 3+ by IHC, and Ki‐67 was measured on the whole stained tumor and categorized as low (<15%) and medium/high (≥15%).

### 2.3 Tumor grade

Tumor grading was performed according to the Nottingham Histologic Score system (Elston grade).

### 2.4 PR intra‐tumor heterogeneity

Intra‐tumor heterogeneity of PR expression was calculated based on the pathologist estimated proportions of tumor cells of each staining intensity level (0, 1+, 2+, 3+) utilizing Rao's quadratic entropy,^26^ in accordance with our previous study assessing ER intra‐tumor heterogeneity.^15^ Rao's quadratic entropy extends the Simpson's index^27^ with the addition of a distance matrix. The distance matrix helps to better capture the differences in PR intensity (Supplementary Methods). As an example, the difference between PR intensity 1+ and 2+ within a tumor would be weighted as 1 and the difference between PR intensity 0 and 3+ within a tumor would be weighted as 3 (Supplementary Methods). A predefined cutoff at the second tertile (67%) for high intra‐tumor heterogeneity was used, as in our previous study on ER heterogeneity,^15^ aiming to identify a smaller subset of tumors with high intra‐tumor heterogeneity. We refer to these categories as high and low intra‐tumor heterogeneity of PR. This aligns with classification of other tumor characteristics, including ER and Ki‐67.

### 2.5 Statistical analyses

The association between the level of PR intra‐tumor heterogeneity and the distribution of tumor characteristics was assessed using Fisher's exact test, for all available characteristics. The clinical endpoint for all analyses was distant recurrence‐free interval (DRFI) with distant recurrence, that is, distant metastatic disease as event.^28^ For two patients with missing date of distant metastasis, the date of death from breast cancer was used. Long‐term risk of distant recurrence by intra‐tumor heterogeneity of PR was assessed using univariate Kaplan–Meier analysis, multivariable Cox proportional hazards regression, and multivariable time‐varying flexible parametric modeling. In the crude and fully adjusted multivariable Cox proportional hazards models and in the multivariable time‐varying flexible parametric model, 504 patients with complete information on all covariates were included, thus excluding (*n* = 16) patients with missing tumor characteristics. The crude model adjusted for age at diagnosis, randomization year, lymph node status (i.e., trial stratification, Figure 1) and type of endocrine therapy. The full model was further adjusted for tumor proliferation (using Ki‐67), HER2‐status, tumor grade and tumor size. Time‐varying flexible parametric modeling was used to estimate time‐varying hazards, aiming to better understand how the impact of PR intra‐tumor heterogeneity on distant recurrence varied over the duration of the follow‐up.^29^ By utilizing splines, the model can accommodate time‐varying coefficients, allowing for non‐proportional hazards. The baseline log‐cumulative hazard was modeled using natural cubic splines with two degrees of freedom, the effect of PR intra‐tumor heterogeneity on DRFI was modeled as a time‐varying coefficient with one degree of freedom using natural cubic splines. Models of varying complexity were evaluated, examining between one to seven degrees of freedom for both parameters. Model selection was performed using the Akaike information criterion and the Bayesian information criterion.

All statistical tests were two‐sided and a *p*‐value less than .05 was considered statistically significant. Analyses were conducted using R version 4.0.5 (package rstpm2 1.6.2, survival 3.5.7 and survminer 0.4.9), Python 3.8.12 and Stata BE 18.0 (package stpm2 1.7.6).

## 3 RESULTS

Out of the 924 premenopausal patients enrolled in the STO‐5 trial, 731 had tumor blocks with sufficient invasive tumor cells. Out of these, 520 patients had ER‐positive/PR‐positive tumors with available PR intra‐tumor heterogeneity data. Patient and tumor characteristics for the ER‐positive and PR‐positive premenopausal patients from the STO‐5 trial are presented (Table 1). Patients with high PR intra‐tumor heterogeneity were significantly more likely to have a higher tumor grade (Fisher's exact test, *p* = .006) and increased tumor cell proliferation (Fisher's exact test, *p* = .005), as measured by Ki‐67 (Table 1).

**TABLE 1:** Patient and tumor characteristics by high versus low PR intra‐tumor heterogeneity in ER‐positive/PR‐positive premenopausal patients.

		Patients by PR intra‐tumor heterogeneity level		Total	*p* ^b^
Patient and tumor characteristics (*n* = 520)^a^		High No (%)	Low No (%)		
Age	<46	72 (41%)	111 (32%)	183	.15
	46–50	77 (44%)	163 (48%)	240	
	>50	28 (16%)	69 (20%)	97	
Endocrine therapy	Goserelin	51 (29%)	92 (27%)	143	.90
	Tamoxifen	40 (23%)	73 (21%)	113	
	Tamoxifen and Goserelin	45 (25%)	90 (26%)	135	
	Control	41 (23%)	88 (26%)	129	
Positive lymph nodes	0	78 (44%)	180 (52%)	258	.15
	1–3	73 (41%)	126 (37%)	199	
	4+	26 (15%)	37 (11%)	63	
Grade	1	20 (11%)	58 (17%)	78	.006
	2	104 (59%)	223 (65%)	327	
	3	53 (30%)	62 (18%)	115	
Tumor size	<20 mm	123 (70%)	232 (69%)	355	.76
	≥20 mm	52 (30%)	106 (31%)	158	
	Unknown	2	5	7	
HER2	Negative	153 (87%)	309 (90%)	462	.24
	Positive	23 (13%)	33 (10%)	56	
	Unknown	1	1	2	
Ki67	Low	111 (63%)	254 (75%)	365	.005
	Med/High	64 (37%)	83 (25%)	147	
	Unknown	2	6	8	
Subtype	Luminal A	75 (53%)	163 (60%)	238	.22
	Luminal B	39 (28%)	63 (23%)	102	
	HER2	13 (9%)	12 (4%)	25	
	Basal	2 (1%)	4 (1%)	6	
	Normal	12 (9%)	30 (11%)	42	
	Unknown	36	71	107	

^a^ In total 520 premenopausal women, out of which 504 had complete information on all tumor characteristics.

^b^ Fisher's exact test was used to compute *p*‐values.

**FIGURE 2:**
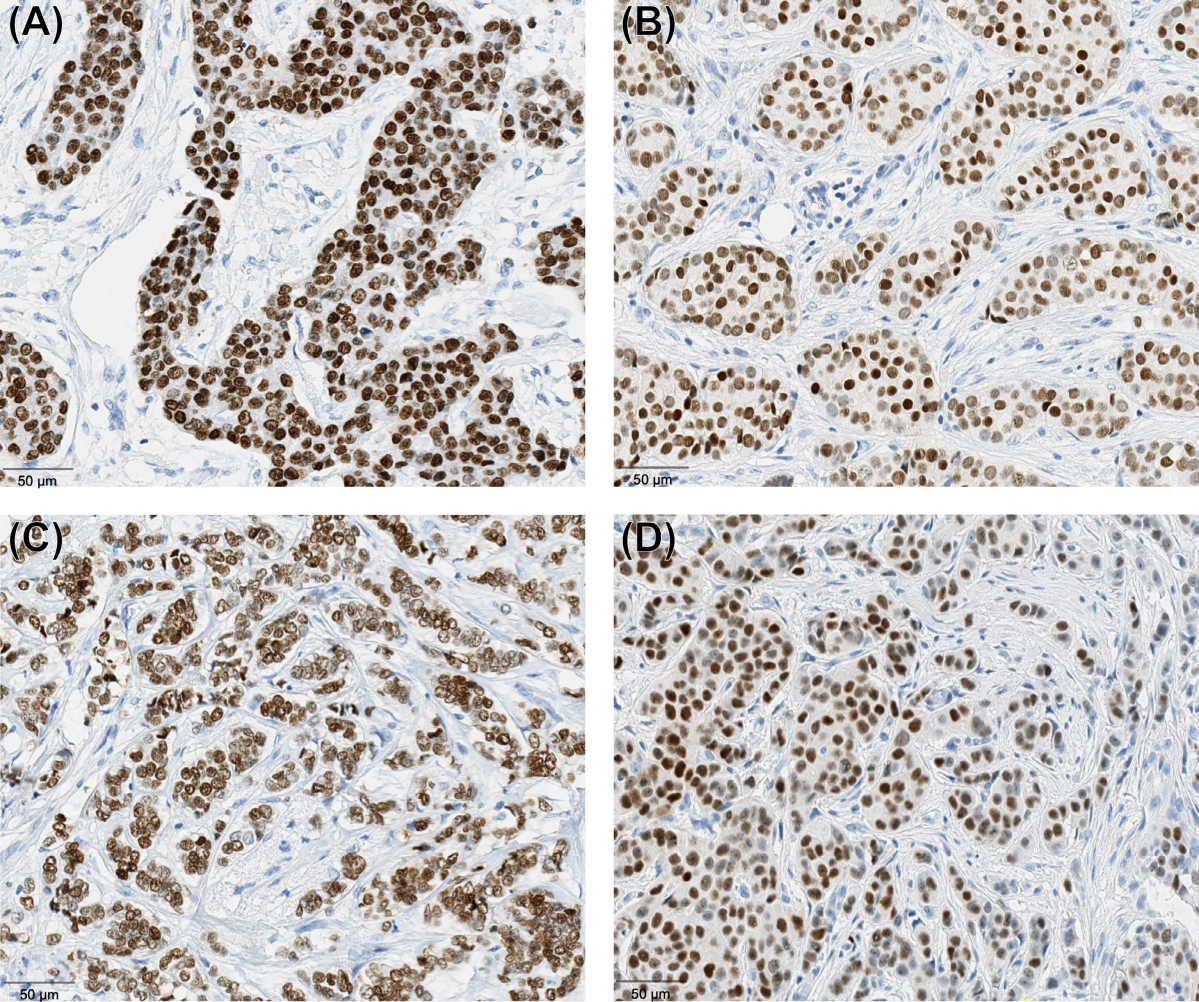
Illustration of low and high PR intra‐tumor heterogeneity in four representative patient tumors. Low intra‐tumor heterogeneity: In (A) PR expression is homogeneous with strong expression, predominantly 3+, and in (C) PR expression is homogeneous at lower intensity, mainly 2 + . High intra‐tumor heterogeneity: In (B) PR expression ranges from strongly positive (3+), to intermediate (2+), and weak (1+), and in (D) PR expression varies from negative, weak (1+), to strong (3+).

IHC images of four representative tumors illustrating low and high intra‐tumor heterogeneity of PR expression intensity are shown (Figure 2). Low intra‐tumor heterogeneity of PR is shown, with strong homogeneous expression (Figure 2A) and intermediate homogeneous expression (Figure 2C). High intra‐tumor heterogeneity of PR is also shown, with PR expression ranging from weak through intermediate to strong (Figure 2B) and including negative, weak, and strong expression (Figure 2D).

### 3.1 DRFI by level of PR intra‐tumor heterogeneity

**FIGURE 3:**
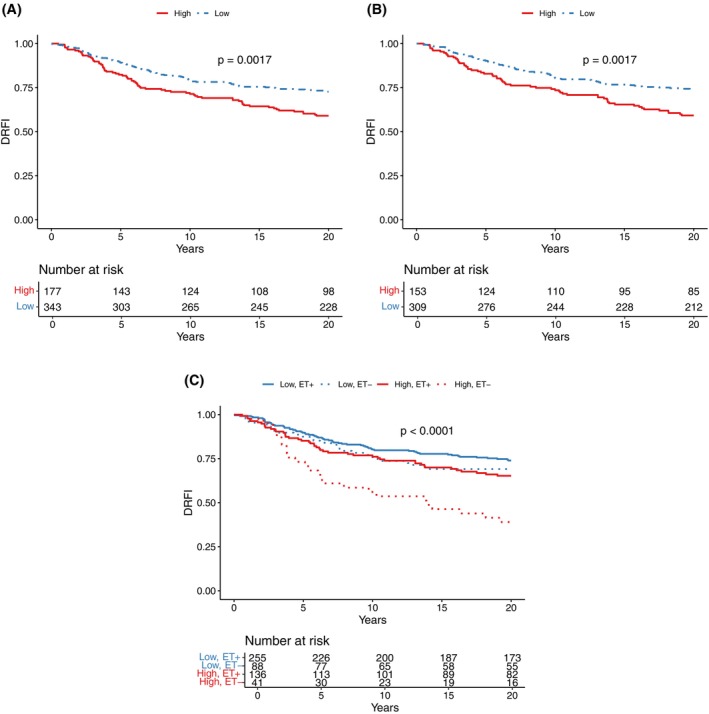
Kaplan–Meier analysis of long‐term risk of distant recurrence, DRFI, by PR intra‐tumor heterogeneity. All patients with ER‐positive/PR‐positive breast cancer in (A), only HER2‐negative ER‐positive/PR‐positive patients in (B), and patients stratified by PR intra‐tumor heterogeneity (low and high) and random assignment to adjuvant endocrine therapy (ET+ or ET−) in (C).

Univariate Kaplan–Meier analysis revealed that patients with high PR intra‐tumor heterogeneity had a significantly shorter DRFI compared to patients with low intra‐tumor heterogeneity (log‐rank *p* = .002) (Figure 3A,B). The survival proportions by DRFI at 20 years were 59% (95% CI, 52%–67%), for patients with high intra‐tumor heterogeneity, and 73% (95% CI, 68%–78%) for patients with low heterogeneity (Figure 3A). Further subgroup analysis restricted to patients with HER2‐negative tumors showed similar results (log‐rank *p* = .002), with 20‐year survival proportions by DRFI of 59% (95% CI, 52%–68%) and 74% (95% CI, 69%–79%) for patients with high and low PR intra‐tumor heterogeneity, respectively (Figure 3B).

Multivariable Cox proportional hazards regression analyses, including patients with complete data on all covariates (*n* = 504), supported the univariate analyses (Table 2). There was a statistically significant increased risk of distant recurrence for patients with high PR intra‐tumor heterogeneity as compared to patients with low heterogeneity (Full model: hazard ratio [HR] = 1.42; 95% CI, 1.02 to 1.96, and Crude model: HR = 1.52; 95% CI, 1.10–2.10). Similar results were seen among patients with HER2‐negative breast cancer; an elevated risk of distant recurrence for patients with high PR intra‐tumor heterogeneity versus low was observed (Full model: HR = 1.43; 95% CI, 1.01–2.02, and Crude model: HR = 1.52; 95% CI, 1.07–2.15).

**TABLE 2:** Multivariable Cox proportional hazards regression analyses of long‐term risk of distant recurrence (DRFI) by PR intra‐tumor heterogeneity and stratified by random assignment to adjuvant endocrine therapy (ET+) or control (ET−).

Cox regression model	Category	Patients	DRs	Crude^a^ HR (95% CI)	Full^b^ HR (95% CI)
PR heterogeneity					
All patients^c^					
PR heterogeneity	High	172	67	1.52 (1.10–2.10)	1.42 (1.02–1.96)
	Low	332	87	1.0 (Ref)	1.0 (Ref)
HER2‐negative subgroup					
PR Heterogeneity	High	150	58	1.52 (1.07–2.15)	1.43 (1.01–2.02)
	Low	301	77	1.0 (Ref)	1.0 (Ref)
Endocrine therapy					
High PR heterogeneity					
Endocrine therapy	ET+	133	44	0.39 (0.23–0.67)	0.41 (0.24–0.71)
	ET−	39	23	1.0 (Ref)	1.0 (Ref)
Low PR heterogeneity					
Endocrine therapy	ET+	246	61	0.69 (0.44–1.10)	0.67 (0.42–1.09)
	ET−	86	26	1.0 (Ref)	1.0 (Ref)

*Note*: DRs = Number of distant recurrences (DRs) that occurred over the 20‐year follow‐up.

^a^ Crude model hazard ratios (HR) with 95% confidence interval. Adjusted for age, randomization year, lymph node status and type of endocrine therapy (not in Endocrine therapy model).

^b^ Full model HRs with 95% confidence interval. Adjusted for age, randomization year, lymph node status, type of endocrine therapy (not in Endocrine therapy model), tumor size, tumor grade, Ki‐67 status, and HER2 status (not in HER2‐negative subgroup).

^c^ Patients with complete information on all covariates (504).

### 3.2 DRFI by PR intra‐tumor heterogeneity and endocrine therapy

We investigated the influence of PR intra‐tumor heterogeneity on DRFI and endocrine therapy benefit. In the Kaplan–Meier analysis, endocrine‐treated patients with low PR intra‐tumor heterogeneity had the most favorable DRFI, 74% (95% CI, 69%–80%), followed by endocrine‐untreated patients with low PR heterogeneity, 69% (95% CI, 60%–80%), and then endocrine‐treated patients with high PR heterogeneity, 65% (95% CI, 58%–74%), (Figure 3C). Notably, the least favorable DRFI was observed in endocrine‐untreated patients with high PR heterogeneity, where the 20‐year DRFI survival proportion declined to 39% (95% CI, 27%–57%).

Multivariable Cox proportional hazards regression analysis among patients with high PR heterogeneity, suggested a significant benefit from endocrine therapy, that is, contrasting endocrine‐treated versus control (Full model: HR = 0.41; 95% CI, 0.24–0.71, Crude model: HR = 0.39, 95% CI 0.23–0.67, Table 2). For patients with low heterogeneity, the benefit from endocrine therapy did not reach statistical significance (Full model: HR = 0.67; 95% CI, 0.42–1.09, Crude model: HR = 0.69; 95% CI, 0.44–1.10).

### 3.3 Time‐varying multivariable analysis of PR intra‐tumor heterogeneity

Time‐varying analysis was conducted to evaluate whether the hazard ratio of distant recurrence for patients with high PR intra‐tumor heterogeneity, compared to those with low heterogeneity, varied over the 20‐year follow‐up period. The analysis suggested an increased long‐term risk for patients with high compared to patients with low PR heterogeneity and was stable throughout the follow‐up. This increased risk was statistically significant between 5 and 12 years, using the full model (Supplementary Figure 1).

## 4 DISCUSSION

Patients with ER‐positive breast cancer have a substantial long‐term risk of distant recurrence, yet the specific tumor characteristics contributing to this long‐term risk remain largely unknown. ER and PR are assessed in clinical routine and are closely interconnected, with PR‐positivity generally indicating a functioning ER signaling pathway. Notably, among patients with ER‐positive tumors, patients that are premenopausal are significantly more likely to also have PR‐positive tumors, compared with their postmenopausal counterparts. Therefore, since PR positivity varies by menopausal status and given the increased risk of fatal disease for premenopausal patients, we investigated whether PR intra‐tumor heterogeneity influences the long‐term risk of distant recurrence. Utilizing the STO‐5 trial, which offers high‐quality 20‐year follow‐up, our findings suggest that premenopausal patients with high PR intra‐tumor heterogeneity have approximately a 40% increased long‐term risk of distant recurrence compared to patients with low intra‐tumor heterogeneity. This association was seen in univariate and multivariable analyses, was consistent in the HER2‐negative subgroup, and persisted over the 20‐year follow‐up. Therefore, assessing PR intra‐tumor heterogeneity may be valuable as an additional marker to more accurately predict long‐term risk of distant recurrence for premenopausal patients.

It is well established that women with ER‐positive tumors have a substantial long‐term risk of recurrence, with a considerable proportion of events occurring beyond 5 years after primary diagnosis. This long‐term risk of recurrence has been shown to remain stable decades later, as documented by us and others,^11^, ^14^, ^30^ and has been suggested to involve tumor cell dormancy. In this state, tumor cells remain viable but non‐dividing, evading effects of treatments specifically targeting proliferating cells, thus enabling metastatic growth at a later point when microenvironmental factors are favorable.^14^ In tumors with a high degree of intra‐tumor heterogeneity, each tumor cell may respond differently to environmental stress, thus increasing the likelihood that some survive, possibly through dormancy, likely making both this mechanism and intra‐tumor heterogeneity important for long‐term risk. Intra‐tumor heterogeneity is also increasingly recognized for its potential role in tumor progression and has also been suggested to influence treatment benefit.^31^, ^32^ Our findings highlight the importance of PR intra‐tumor heterogeneity in understanding the long‐term risk of recurrence in premenopausal breast cancer patients.

In this study, premenopausal patients with high PR intra‐tumor heterogeneity had an increased risk of distant recurrence, but our results did not indicate a reduced benefit from endocrine therapy for these patients. Further, patients with low intra‐tumor heterogeneity treated with endocrine therapy had the lowest long‐term risk. Notably, tumors with high PR intra‐tumor heterogeneity were associated with a higher tumor grade. This suggests an association between intra‐tumor heterogeneity and more aggressive tumor characteristics. Potentially, the observed relationship between poor differentiation and elevated intra‐tumor heterogeneity could be influenced by tumor cell diversity during tumor progression, impacting both intra‐tumor heterogeneity and histological grade.^33^ The link between elevated proliferation and increased PR intra‐tumor heterogeneity might be intertwined with its relation to tumor grade.

There are limitations to this study. A limitation with long‐term follow‐up studies is evolving clinical management. HER2 targeted treatments like trastuzumab have been introduced since the inception of the trial, and we therefore also conducted analyses including only HER2‐negative patients. These analyses corroborated our primary findings including all ER‐positive/PR‐positive patients. Due to the limited number of patients with HER2‐positive tumors (*n* = 56) our conclusions are mainly applicable for HER2‐negative patients. Clinical management of metastatic disease has improved during the trial. We accounted for this by selecting DRFI over an endpoint such as breast cancer specific survival. Also, endocrine therapy today is generally administered at lower dosage but given over an extended duration compared to this trial. Although 520 ER‐positive/PR‐positive premenopausal patients were included in the analyses, sample size is a limitation in some subanalyses and caution should therefore be taken in the interpretation. Recognizing the variability in IHC assessments, we standardized the staining processes across one laboratory and preferred global scoring over hotspot scoring for Ki‐67 to ensure consistency. To aid with scoring of the proportion of tumor cells for each PR staining intensity pathologists underwent training together, discrepancies in scoring were resolved by consensus review.

In conclusion, our findings demonstrate that PR intra‐tumor heterogeneity is associated with an increased long‐term risk of recurrence in premenopausal patients with ER‐positive and PR‐positive breast cancer. This association was consistent within the HER2‐negative subgroup and remained stable over the 20‐year follow‐up period. Patients with high PR heterogeneity had a significant benefit from endocrine therapy, indicating that endocrine therapy remains effective despite PR heterogeneity. These results suggest that assessing PR intra‐tumor heterogeneity may enhance long‐term risk assessment for premenopausal breast cancer patients and could be valuable as an additional marker in clinical practice for personalized treatment strategies. In an ongoing study, we are using highly multiplexed imaging mass cytometry to characterize the tumor microenvironment at single‐cell spatial resolution, which may clarify how intra‐tumor heterogeneity of PR and other markers influences the tumor microenvironment and long‐term risk.

## AUTHOR CONTRIBUTIONS

**Oscar Danielsson:** Conceptualization; data curation; methodology; software; validation; investigation; formal analysis; visualization; writing – original draft; writing – review and editing. **Huma Dar:** Validation; data curation; formal analysis; investigation; software; visualization; writing – review and editing. **Gizeh Perez‐Tenorio:** Writing – review and editing. **Anna Nordenskjöld:** Writing – review and editing. **Christina Yau:** Writing – review and editing. **Christopher C. Benz:** Writing – review and editing. **Laura J. Esserman:** Writing – review and editing. **Bo Nordenskjöld:** Project administration; resources; writing – review and editing. **Olle Stål:** Writing – review and editing; project administration; resources. **Tommy Fornander:** Project administration; resources; writing – review and editing. **Johan Hartman:** Writing – review and editing. **Nicholas P. Tobin:** Writing – review and editing. **Annelie Johansson:** Writing – review and editing; conceptualization; data curation. **Linda S. Lindström:** Conceptualization; data curation; formal analysis; funding acquisition; investigation; methodology; project administration; resources; software; supervision; writing – review and editing; writing – original draft.

## FUNDING INFORMATION

This work was supported by the Swedish Research Council [Vetenskapsrådet, grant number 2020‐02466 and 2023‐03009 to L.S.L.]; ALF Medicine [grant number FoUI‐974882 to L.S.L.]; the Gösta Milton Donation Fund [Stiftelsen Gösta Miltons donationsfond, to L.S.L.]; the Swedish Cancer Society [Cancerfonden, grant number 222216 to O.S., 232670 to N.P.T., and 222081 and 220552SIA to L.S.L.]; Stockholm Cancer Society [Cancerföreningen i Stockholm, grant number 181093 to T.F., 224112 to N.P.T., and 221233 and 201212 to L.S.L.]; King Gustav V Jubilee Clinical Research Foundation [grant number 2021‐356 to A.N.]. The funders had no role in the design of the study; in the collection, analyses, or interpretation of data; in the writing of the manuscript; or in the decision to publish the results.

## CONFLICT OF INTEREST STATEMENT

Coauthor J.H. has obtained speaker's honoraria or advisory board remunerations from Gilead, Novartis, Pfizer, Eli Lilly, MSD, AstraZeneca, Sakura, Hologic and has received institutional research support from MSD and AstraZeneca. J.H. is co‐founder and shareholder of Stratipath AB. All other authors declare no conflicts of interest.

## ETHICS STATEMENT

The trial was approved by the Regional Ethics Committee at Karolinska Institutet. Informed consent was obtained before random assignment. The trial was conducted before the custom of trial registration in Sweden.

## Supporting information


**Data S1:** Supporting Information

## Data Availability

Restrictions apply to the availability of these data according to GDPR. Data were obtained from the STO Trialist Group and are available from the authors with the permission from the STO Trialist Group.
