# Supplementary material for: Progesterone receptor (PR) intra‐tumor heterogeneity in premenopausal breast cancer: A secondary analysis of a randomized trial
Source: Int J Cancer. 2025 Oct 24;158(4):1106–15. doi: 10.1002/ijc.70209 (PMC12712363; doi:10.1002/ijc.70209)
Supplement: Supplementary file 1 — Data S1: Supporting Information [file IJC-158-1106-s001.pdf]

Supplementary Online Content

Progesterone Receptor (PR) Intra-tumor Heterogeneity in Premenopausal Breast Cancer: A Secondary Analysis of a Randomized Trial

Oscar Danielsson, Huma Dar, Gizeh Perez-Tenorio, Anna Nordenskjöld, Christina Yau, Christopher C. Benz, Laura J. Esserman, Bo Nordenskjöld, Olle Stål, Tommy Fornander, Johan Hartman, Nicholas P. Tobin, Annelie Johansson, and Linda S. Lindström

Supplementary Methods 1. Details regarding randomization to endocrine therapy in the STO-5 trial.

Supplementary Methods 2. Intra-tumor heterogeneity of PR.

Supplementary Table 1. Comparison of patient and tumor characteristics for patients analyzed with all STO-5 patients.

Supplementary Figure 1. Time-varying analyses of PR intra-tumor heterogeneity.

This supplementary material has been provided by the authors to give readers additional information about their work.

Supplementary Methods 1. Details regarding randomization to endocrine therapy in the STO-5 trial.

The Stockholm Tamoxifen Trial (STO-5), part of the Zoladex in Premenopausal Patients (ZIPP) initiative, enrolled premenopausal patients with invasive operable breast cancer between May 1990 to January 1997. Eligibility criteria excluded patients with distant metastasis at diagnosis, prior exposure to radiotherapy and endocrine therapy. Enrolled patients did not receive neoadjuvant chemotherapy or presurgical radiotherapy. Surgical procedure for the participants included either full mastectomy or breast-conserving surgery followed by post-operative local breast radiotherapy.

Patients were randomized into four treatment arms: goserelin (3.6 mg subcutaneously every 28 days), tamoxifen (40 mg orally daily), a combination of both, or no adjuvant endocrine therapy (control). Randomization was stratified by number of positive lymph-nodes and categorized into three groups: none, 1-3 positive, 4 or more positive lymph nodes. Patients were randomized into the groups through a central regional office using permuted block randomization, ensuring balanced groups. Once randomization was registered, the treatment assignment was revealed to the clinician.

Lymph node-positive patients underwent six cycles of CMF chemotherapy (cyclophosphamide 500 mg/m^2, methotrexate 40 mg/m^2, fluorouracil 600 mg/m^2) administered intravenously on days 1 and 8 of each 28-day cycle. Additionally, patients with four or more positive lymph nodes received locoregional radiotherapy (46 Gy over 4.5 weeks, targeting the chest wall, axillary and supraclavicular lymph nodes). Follow-up protocols included clinical checks every three months for the initial two years, every six months for the following three years, and then annually.

Supplementary Methods 2. Intra-tumor heterogeneity of PR

Intra-tumor heterogeneity of PR expression was assessed using Rao's quadratic entropy. This method is particularly well-suited for analyzing intra-tumor heterogeneity of staining intensity since it incorporates a distance matrix *D*, weighting differences in staining intensity.

For each tumor the proportion of tumor cells at each PR staining intensity level (0, 1+, 2+, 3+) was scored by pathologists. PR intra-tumor heterogeneity was computed by pairwise multiplication of the proportion of tumor cells at each intensity level, Equation 1.1, taking into account the difference in staining intensity levels as weighted by the distance matrix spanning from 0 (same intensity level) to 3 (difference between 0 to 3+), Equation 1.2. The distance matrix *D* weights how similar any two staining intensities *i* and *j* are to each other. Larger differences in staining intensity are given a higher weight.

$$\sum_{i=0}^{3}\sum_{j=0}^{3} d_{ij} p_i p_j \qquad (1.1)$$

$$D = \begin{bmatrix} 0 & 1 & 2 & 3 \\ 1 & 0 & 1 & 2 \\ 2 & 1 & 0 & 1 \\ 3 & 2 & 1 & 0 \end{bmatrix} \qquad (1.2)$$

Equation 1.1: Rao's quadratic entropy.

Equation 2.2: Definition of the distance matrix

Supplementary Table 1. Comparison of the distribution of patient and primary tumor characteristics for the original STO-5 patients (N=924) and the patients with annotated breast cancer markers (N=731)

Primary patient and tumor characteristics		All STO-5 patients (N=924) No (%)	Patients available for analysis (N=731) No (%)	P[a]
**Age**	<46	326 (35%)	239 (33%)	0.51
	46-50	453 (49%)	368 (50%)	
	>50	145 (16%)	124 (17%)	
**Tumor size**	<20mm	592 (65%)	465 (65%)	0.95
	≥20mm	317 (35%)	255 (35%)	
	Unknown	15 (-)	11 (-)	
**Positive lymph nodes**	0	465 (50%)	364 (49.8%)	0.87
	1-3	323 (35%)	264 (36.1%)	
	4+	136 (15%)	103 (14.1%)	
**Trial arm**	Goserelin	230 (25%)	194 (27%)	0.79
	Tamoxifen	231 (25%)	169 (23%)	
	Tamoxifen and Goserelin	230 (25%)	185 (25%)	
	Control	233 (25%)	183 (25%)	

[a] Fisher's exact test was used to compute p-values

Supplementary Figure 1. Multivariable time-varying flexible parametric modeling of long-term risk of distant recurrence (DRFI) by PR intra-tumor heterogeneity In **A,** hazard rates are shown, and in **B,** the corresponding hazard ratio is plotted with a 95% confidence interval (gray). Dark gray indicates a statistically significant difference.

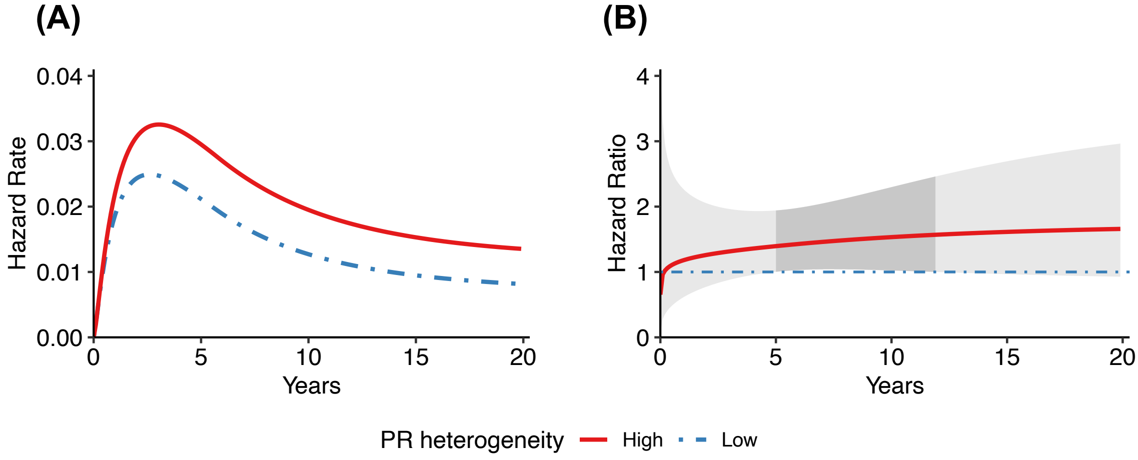

Adjusted for age, randomization year, lymph node status, type of endocrine therapy, proliferation (Ki-67), HER2 status, tumor size and tumor grade. Model selection was done using Akaike information criterion (AIC) and Bayesian information criterion (BIC). Two degrees of freedom were used for the baseline hazard function and PR intra-tumor heterogeneity was modeled as a time-varying coefficient with one degree of freedom.
